# Lifestyle Advice and Self-Care Integral to Acupuncture Treatment for Patients with Chronic Neck Pain: Secondary Analysis of Outcomes Within a Randomized Controlled Trial

**DOI:** 10.1089/acm.2016.0303

**Published:** 2018-03-27

**Authors:** Hugh MacPherson, Ben Elliot, Ann Hopton, Harriet Lansdown, Stephen Birch, Catherine Hewitt

**Affiliations:** ^1^ Department of Health Sciences, University of York, York, United Kingdom.; ^2^ Northern College of Acupuncture, York, United Kingdom.; ^3^ Department of Health Sciences, Kristiania University College, Oslo, Norway.

**Keywords:** acupuncture therapy, traditional East Asian medicine, chronic neck pain, lifestyle advice, self-care

## Abstract

***Background:*** Lifestyle advice is widely considered as an integral component of acupuncture treatment. However, it is unclear whether lifestyle advice and related self-care are important for sustaining benefit over the longer term. In a novel secondary analysis of trial data, this paper explores the nature and impact of acupuncture-related diagnosis, and associated lifestyle advice and self-care, in patients with chronic neck pain.

***Design:*** In a three-arm, randomized, controlled multicenter trial with 12 months of follow-up, a total of 517 patients with chronic neck pain were randomized in equal proportions to acupuncture, Alexander technique, or usual care alone.

***Methods:*** For each acupuncture patient, practitioners reported treatment components that included an acupuncture-related diagnosis and provision of associated lifestyle advice. Patients reported at baseline, 3, 6, and 12 months on variables related to treatment, which included aspects of self-care, self-efficacy, and lifestyle advice acted upon, as well as pain and disability scores. Congruence between practitioner advice and patient take-up was assessed using chi-squared test. Impact of lifestyle advice and self-efficacy on outcome was evaluated using regression models.

***Results:*** Among patients randomized to acupuncture, the most common diagnostic framework involved the Zang–Fu syndromes for 139/160 (87%) patients. Lifestyle advice was provided by practitioners to 134/160 (84%) of patients, most commonly related to exercise, relaxation, diet, rest, and work. Significant congruence with patient take-up was found for diet, rest, and work. Moreover, patients in the acupuncture group improved their ability to use what they had learnt and increased their self-efficacy. In turn, these characteristics were associated with significant reductions in pain and disability scores at 12 months.

***Conclusion:*** Acupuncture-related lifestyle advice helped patients improve the way they live and care for themselves and enhanced self-efficacy and ability to use what they had learnt. These changes were associated with reductions in pain and disability at 12 months.

## Introduction

Acupuncture has been characterized as primarily involving the insertion of needles. In traditional East Asian medicine, acupuncture is usually a more complex intervention because treatment involves multiple components that are guided by the same overarching theoretical perspective, which is designed to impact on outcome.^1^ As well as needling, these components include the diagnostic process and the related explanations and lifestyle advice to promote and enhance patient self-care.

As a consequence, lifestyle advice is widely considered as being integral to treatment by acupuncture practitioners who use the principles of traditional East Asian medicine in routine practice. For example, in the United Kingdom, lifestyle advice relevant to an acupuncture diagnosis is provided by practitioners to 56% of their patients, which rises to 85% of patients of practitioners who provide a predominantly traditional Chinese medical style of treatment.^2^ In Europe and China, acupuncture advice provided by practitioners is primarily related to diet (73% and 67%, respectively) and exercise (50% and 49%).^3^ Further studies have elaborated on the role of lifestyle advice and self-care within traditional acupuncture consultations^4^ and the coconstruction of self-care within the context of the therapeutic relationship in a way that is different from that of a conventional medical consultation.^5^

The question as to whether the lifestyle advice and related self-care actually do impact in terms of helping promote longer-term benefit is important and needs addressing. This question is relevant to the search for explanations of long-term benefits that have been reported in trials of traditional acupuncture, within which the provision of lifestyle advice as a treatment component has been supported. Qualitative data related to the trial of acupuncture for low back pain^6^ have suggested the importance of lifestyle advice^7,8^ as well as from trials for irritable bowel syndrome^9,10^ and for depression.^11,12^ What is needed, however, are quantitative data to establish whether lifestyle advice, when it is an integral component of acupuncture treatment, enhances overall benefit.

This paper expands on the primary results of the ATLAS (Alexander Technique Lessons or Acupuncture Sessions) chronic neck pain trial published previously.^13^ In a population that had experienced chronic neck pain for on average 6 years, we found that there were statistically significant benefits at 6 and 12 months for patients who received either Alexander technique plus usual care or acupuncture plus usual care when compared with those receiving usual care alone. We also found that the self-efficacy of patients in both the acupuncture and the Alexander technique arms improved when compared with usual care at 6 months. Improved patient self-efficacy meant that patients learned and used better strategies for reducing pain without resorting to medication. Moreover, this improvement in self-efficacy was associated with significant reductions in neck pain at 6 and 12 months.

There is a need to better understand these longer-term benefits and whether lifestyle advice translates into better self-care and improved self-efficacy. In prespecified secondary analyses, our aim in this paper is to explore whether there were acupuncture-specific diagnostic variables or lifestyle-related factors that might help explain the reported longer-term benefits at 6 and 12 months.

## Methods

### Trial design

The design involved a pragmatic three-arm trial with patients randomized in equal proportions to acupuncture, to Alexander technique, or to usual medical care alone. All patients continued to receive usual medical care throughout the trial. Patients were recruited from primary care by first identifying potential participants from GP databases. Patients were invited to return baseline questionnaires and consent forms, which were then screened for eligibility by members of the research team at the University of York. A minimum score of 28% was required on the Northwick Park neck pain and disability questionnaire (NPQ),^14^ a percentage-based scale, which was our primary outcome measure. A total of 517 patients were recruited and randomized between March 2012 and April 2013.

### Acupuncture treatment

Those randomized to acupuncture were offered up to 12 sessions and those randomized to Alexander technique were offered up to 20 sessions. To be eligible to provide treatments within the trial, acupuncture practitioners were required to be members of the British Acupuncture Council with at least 3 years postqualification experience and a commitment to continuing professional development. Moreover, they agreed to provide a style of acupuncture based on traditional Chinese medicine principles, the most commonly used approach to the provision of acupuncture in the United Kingdom.^2^ As this was a pragmatic clinical trial, practitioners were encouraged to practice as closely as possible to how they would routinely.^15^ Acupuncture practiced in this way can be considered a complex intervention,^1^ and integral to treatment were the acupuncture diagnosis-related components, such as explanations of the diagnosis and diagnosis-specific lifestyle advice.^7^

### Data collected from practitioners at the end of treatment

Practitioners documented in their logbook data for each patient: their acupuncture-related diagnosis; the points used each session; the auxiliary modalities provided; and the lifestyle advice given over the course of treatment. Using the eight-principle framework, practitioners identified whether the neck pain was an external, an internal, or a mixed internal and external condition. Practitioners assessed whether their patients could be diagnosed as predominantly having had one of two common clusters of Zang–Fu syndromes: predominantly Liver-related syndromes that included Liver Qi Stagnation, Liver Yin Xu, Liver Yang Rising, and Liver Fire and predominantly Spleen-related syndromes that included Spleen Qi and Yang Xu, Kidney Qi and Yang Xu, and Internal Damp. Clustering in this way helped when conducting subsequent analyses. Moreover, practitioners provided a rationale for any lifestyle advice related to acupuncture theory.

### Data collected from participants at baseline, 6, and 12 months

At baseline, data were collected for each participant on a number of factors, including the duration of their neck pain, age, gender, and home city, as well as GP practice. At baseline, 6, and 12 months, we also assessed patients' perceived stress levels using the four-question Perceived Stress Scale, each item scored 0 to 4 with total scores 0 to 16.^16^ At baseline, 6, and 12 months, self-efficacy was determined by the 5-question pain management subscale of the Chronic Pain Self-Efficacy Scale scored 0 to 8.^17^ At 6 and 12 months, data were collected on self-care, the extent that it took place, and the extent that it was perceived to be beneficial. Patients responded to the question, “Can you use/apply the things you learned from the treatment/care in everyday life situations to reduce pain?” with responses as never, seldom, occasionally, often, and every day (scored 0 to 4, respectively). Patients responded to the question, “To what extent are you able to put into practice the advice or teaching you received?” on an 11-point scale of increments of 1 from 0 (not at all) to 10 (completely). Using the same 11-point scale, patients responded to the question, “To what extent are the changes you have been making helpful to you?” Patients responded (yes/no) to the question, “During the treatment/care you received in the last 6 months, did you learn to improve the way you live and care for yourself?” Patients also reported whether they made changes related to diet, exercise, relaxation, rest, and work.

### Analyses

Analyses were conducted in Stata, version 13, using two-sided significance tests at the 5% significance level (StataCorp. 2013. *Stata Statistical Software: Release 13*. College Station, TX: StataCorp LP). All comparisons of acupuncture and usual care were conducted on an intention-to-treat basis, including all patients in the groups to which they were randomized. Descriptives are reported as means and standard deviations for continuous variables and counts and percentages for categorical variables.

The associations between practitioner-reported advice and patient-reported lifestyle changes were also explored using chi-squared tests. The eight principles and Zang–Fu clusters, and their relationship with NPQ change scores at 6 and 12 months, are presented as means with 95% confidence intervals.

The ability to make improvements in living/self-care and changes in diet, exercise, relaxation, rest, and work at 6 and 12 months were analyzed individually by logistic regression. Patient-reported perceived stress, self-efficacy, ability to use what has been learnt, number of lifestyle changes, extent put into practice advice or teaching, and extent to which changes were helpful at 6 and 12 months were analyzed individually by linear regression. To explore the impact of patient-reported variables measured during the intervention period and changes in NPQ outcomes at 6 and 12 months, linear regression was utilized. NPQ outcomes were analyzed individually at each time point and included the patient-reported variables (or changes in these variables from baseline) as fixed effect covariates in the model. All regression analyses made adjustments for baseline NPQ scores, duration of neck pain, age, gender, and city as a fixed effect, and GP practice as a random effect using robust standard errors (Stata *regress* command with cluster option).

## Results

Of the 173 participants randomized to acupuncture, 162 (94%) received at least one treatment, 125 (72%) attended all 12 sessions, and 11 (6%) did not attend any treatment. Of the 162 patients attending at least one treatment, 2 withdrew from the trial. A total of 1,775 treatments were received, and the average number of sessions attended was 11 (range = 1 to 12, median 12). Treatments were provided by 18 acupuncture practitioners (15 females), each of whom treated on average 10 patients (range 2 to 22). The acupuncture points used and auxiliary treatments provided are documented in the Supplementary Data (Supplementary Data are available online at www.liebertpub.com/acm). Other components of treatment are presented below.

### Traditional acupuncture diagnosis

Practitioners were asked to record their methods of diagnosis within a traditional Chinese medicine theoretical framework (Table 1). The three predominant frameworks used were Zang–Fu (139/160; 87%), fundamental substances (117/160; 73%), and eight principles (94/160; 59%). Using the eight principles, practitioners documented their assessment on whether their patient's neck pain could be diagnosed as more external, more internal, or a mixed internal and external condition. Most commonly, practitioners reported it to be a mixed internal and external condition (86/160; 54%). For patients diagnosed as having a Zang–Fu disharmony, practitioners categorized the syndromes in the majority of their patients as either predominantly Liver related (84/160; 53%), followed by Spleen (53/160; 33%), and neither (2/160; 1%).

**Table 1.** Theoretical Frameworks Used for Acupuncture-Specific Diagnosis

*Traditional acupuncture diagnosis*	*Frequency (*n* = 160),* n *(%)*
Overall theoretical framework	
Zang–Fu syndromes	139 (86.9)
Fundamental substances	117 (73.1)
Eight principles	94 (58.8)
Pathogenic factors	36 (22.5)
Eight extravessels	17 (10.6)
Four levels	4 (2.5)
Six divisions	3 (1.9)
Five elements	3 (1.9)
Other	3 (1.9)
Eight-principle diagnosis	
Mixed internal and external	86 (53.8)
Predominantly external	41 (25.6)
Predominantly internal	33 (20.6)
Zang–Fu syndrome clusters	
Liver cluster: Liver Qi Stagnation, Liver Yin Xu, Liver Yang Rising and Liver Fire	84 (52.5)
Spleen cluster: Spleen Qi and Yang Xu, Kidney Yang Xu, and Internal Damp	53 (33.1)
Neither of the above two clusters	2 (1.5)

Practitioners reported up to five key symptoms that guided their diagnosis of either the Liver or Spleen clusters. Within the Liver cluster, we found the most commonly reported symptoms to be stress (38/84; 45%), muscular tension/stiffness (31/84; 37%), and headache/migraine (28/84; 33%) (Table 2). Within the Spleen cluster, we found the most commonly reported symptoms to be pain (33/53; 62%) and tiredness/fatigue (16/53; 30%).

**Table 2.** Most Commonly Reported Symptoms in Those Diagnosed with Predominantly Liver or Spleen Cluster Pathology

*Rank*	*Symptom reported for patients with Liver cluster pathology (*n* = 84)*	*No. of times,* n *(%)*	*Symptom reported for patients with Spleen cluster pathology (*n* = 53)*	*No. of times,* n *(%)*
1	Stress	38 (45.2)	Pain	33 (62.3)
2	Muscular tension/stiffness	31 (36.9)	Tiredness/fatigue	16 (30.2)
3	Headache/migraine	28 (33.3)	Bloating	5 (9.4)
4	Pain	26 (31.0)	Cold aversion	5 (9.4)
5	Sleep disturbances/insomnia	15 (17.9)	Cold extremities	5 (9.4)
6	Irritability	14 (16.7)	Heavy limbs	5 (9.4)
7	Anger	11 (13.1)	Digestive problems	4 (7.5)
8	Depression	7 (8.3)	Headache/migraine	4 (7.5)
9	Frustration	4 (4.8)	Edema	4 (7.5)
10	Menstrual irregularities	4 (4.8)	Overweight	4 (7.5)
11	Red face	4 (4.8)	Low bone density	3 (5.7)
12	Anxiety	3 (3.6)	Poor appetite	3 (5.7)
13	Fluctuating mood	3 (3.6)	Prolapse	3 (5.7)

### Practitioner-reported advice offered to patients based on the acupuncture diagnosis

Within the protocol for treatment and integral to the provision of acupuncture, practitioners were encouraged to provide lifestyle advice where appropriate, provided that it was linked to traditional acupuncture theory and the patient's diagnosis. Practitioners reported in the logbooks that they gave advice to make lifestyle changes to 134 (134/160; 84%) patients in total. This was most commonly related to advice about exercise (72/134; 54%), relaxation (59/134; 44%), diet (54/134; 40%), rest (47/134: 35%), work (31/134; 23%), and other advice (29/134; 22%).

The practitioners also reported in the logbooks the reasons for the advice based on traditional acupuncture theories. Advice related to exercise was most commonly to move the Liver Qi Stagnation (67/72; 93%). Advice related to relaxation was not only predominantly to move the Liver Qi (33/59; 56%) but also to strengthen Spleen/Kidneys (12/59; 20%). Dietary advice was predominantly not only to strengthen the Spleen and resolve Damp (34/54; 63%) but also to tonify the Yin and Blood (17/54; 31%). Advice regarding work was commonly focused on moving the Liver Qi (16/31; 52%). The other advice was often to protect the channels from external pathogenic factors and move channel stagnation (17/29; 59%), and also to advise on a referral (8/29; 28%).

### Relationship between the advice given by a practitioner and whether the advice was acted upon by the patient

We compared data related to advice from practitioner logbooks with that reported by patients in questionnaires at 6 and 12 months. We found some evidence of a significant or near-significant congruence between advice reported by practitioners and lifestyle changes reported by patients at 12 months related to diet and rest and work (Table 3).

**Table 3.** Congruence Between Advice Given (Practitioner Data) and Advice Taken Up (Patient Data)

		*Patients reported making changes (Yes/No)*						
		*6 months*				*12 months*		
*Practitioner advice given*	*Yes/No*	*Yes*	*No*	*Chi-squared*		*Yes*	*No*	*Chi-squared*
Diet	Yes	12 (23.1)	40 (76.9)	0.88	Yes	14 (27.5)	37 (72.6)	0.07
	No	18 (22.0)	64 (78.1)		No	11 (14.3)	66 (85.7)	
		Yes	No			Yes	No	
Exercise	Yes	27 (38.0)	44 (62.0)	0.12	Yes	24 (34.8)	45 (65.2)	0.92
	No	16 (25.4)	47 (74.6)		No	20 (33.9)	39 (66.1)	
		Yes	No			Yes	No	
Relaxation	Yes	22 (38.6)	35 (61.4)	0.28	Yes	20 (35.7)	36 (64.3)	0.25
	No	37 (48.0)	40 (52.0)		No	33 (45.8)	39 (54.2)	
		Yes	No			Yes	No	
Rest	Yes	12 (26.7)	33 (73.3)	0.46	Yes	8 (18.6)	35 (81.4)	0.06
	No	29 (33.0)	59 (67.1)		No	29 (34.5)	55 (65.5)	
		Yes	No			Yes	No	
Work	Yes	14 (45.2)	17 (54.8)	0.002	Yes	11 (37.9)	18 (62.1)	0.003
	No	18 (17.8)	83 (82.2)		No	13 (13.4)	84 (86.6)	

### Relationship between traditional acupuncture medical diagnosis and outcome

The relationship between practitioner-reported traditional acupuncture medical diagnoses and patient-reported changes in pain (NPQ) scores at 6 and 12 months is presented in Table 4. Most notably, the mean NPQ change scores were larger (with larger negative reductions indicating better outcomes) at both 6 and 12 months when the neck pain was classified as predominantly external (a channel only problem) compared with predominantly internal (a Zang–Fu problem) or with mixed internal and external problem (involving both channel and Zang–Fu).

**Table 4.** Eight Principles and Zang–Fu Clusters: Relationship to NPQ Change Scores at 6 and 12 Months

	*NPQ change scores between baseline and 6 months*		*NPQ change scores between baseline and 12 months*	
*Acupuncture-related diagnostic methods*	N *(%)*	*Mean (95% CI)*	N *(%)*	*Mean (95% CI)*
Eight principles^a^				
External	41 (27)	−15.33 (−20.15 to −10.51)	37 (25)	−20.29 (−24.98 to −15.60)
Internal	30 (20)	−13.37 (−18.98 to −7.75)	29 (20)	−8.41 (−14.15 to −2.66)
Mixed internal and external	82 (54)	−10.58 (−13.32 to −7.84)	80 (55)	−10.55 (−13.31 to −7.78)
Zang–Fu clusters^b^				
Liver cluster	82 (62)	−12.29 (−15.06 to −9.52)	76 (60)	−10.32 (−13.45 to −7.18)
Spleen cluster	48 (36)	−9.97 (−14.49 to −5.45)	48 (38)	−11.30 (−15.30 to −7.30)
Other^c^	2 (2)	—	2 (2)	—

^a^ Missing data: 3 internal and 4 mixed internal and external patients did not provide NPQ scores at 6 months; and 4 external, 4 internal, and 6 mixed internal and external patients did not provide NPQ scores at 12 months.

^b^ Missing data: 2 Liver and 5 Spleen cluster patients did not provide NPQ data at 6 months; and 8 Liver and 5 Spleen cluster patients did not provide NPQ data at 12 months.

^c^ Insufficient data for analysis.

CI, confidence interval; NPQ, Northwick Park neck pain and disability questionnaire.

### Self-care and lifestyle when comparing acupuncture and usual care groups at 6 and 12 months

The results of the regression analyses are presented in Table 5. There was evidence of a difference between acupuncture and usual care at 12 months in patient-reported self-efficacy, the number of lifestyle changes made, the ability to make improvements in living/self-care, and in making specific lifestyle changes related to diet, exercise, relaxation, resting, and work.

**Table 5.** Patient-Reported Variables Comparing Acupuncture and Usual Care Groups at 6 and 12 Months

	*6 months*				*12 months*			
*Characteristic*	*Acupuncture* N*, mean (SD)*	*Usual care alone* N*, mean (SD)*	*Difference (95% CI)*	p	*Acupuncture* N*, mean (SD)*	*Usual care alone* N*, mean (SD)*	*Difference (95% CI)*	p
Chronic Pain Self Efficacy Scale (scored 0 to 8)	151, 4.80 (1.80)	139, 3.92 (1.52)	0.82 (−0.49 to 1.16)	<0.001	144, 4.88 (1.79)	139, 4.14 (1.68)	0.65 (0.18 to 1.13)	0.009
Perceived Stress Scale (scored 0 to 16)	151, 5.85 (3.29)	144, 5.67 (3.23)	0.14 (−0.58 to 0.87)	0.69	145, 5.92 (3.43)	140, 5.84 (3.48)	0.11 (−0.53 to 0.75)	0.74
“Can you use/apply the things you learned from the treatment in everyday life situations to reduce pain?”(0 to 4)	147, 1.75 (1.21)	125, 1.45 (1.11)	0.29 (−0.01 to 0.60)	0.06	144, 1.70 (1.16)	128, 1.48 (1.09)	0.23 (−0.06 to 0.52)	0.12
Number of lifestyle changes (1 to 5)	156, 1.46 (1.50)	148, 0.41 (0.86)	1.04 (0.73 to 1.36)	<0.001	150, 1.38 (1.36)	144, 0.58 (1.13)	0.81 (0.55 to 1.07)	<0.001
“To what extent are you able to put into practice the advice you received?”(0 to 10)	107, 5.68 (3.13)	43, 4.28 (3.20)	1.52 (0.48 to 2.55)	0.005	104, 5.46 (2.80)	44, 5.16 (3.26)	0.30 (−0.95 to 1.55)	0.63
To what extent are the changes you have been making helpful to you?(0 to 10)	106, 5.77 (3.07)	45, 4.29 (2.98)	1.52 (0.53 to 2.50)	0.004	103, 5.76 (2.75)	46, 4.91 (2.95)	0.82 (−0.49 to 2.12)	0.21

	*6 months*				*12 months*			
*Characteristic (Yes/No responses)*	*Acupuncture*	*Usual care alone*	*Odds ratio (95% CI)*	p	*Acupuncture*	*Usual care alone*	*Odds ratio (95% CI)*	p
“Did you learn to improve the way you live and care for yourself?”	87/149 (58.4)	29/128 (22.7)	5.35 (3.47 to 8.23)	<0.001	90/144 (62.5)	31/123 (25.2)	5.39 (3.32 to 8.74)	<0.001
Did you make any changes to	*N* = 156	*N* = 148			*N* = 150	*N* = 144		
Diet	32 (20.5)	8 (5.4)	5.32 (2.29 to 12.32)	<0.001	28 (18.7)	13 (9.0)	2.74 (1.45 to 5.21)	0.002
Exercise	48 (30.8)	27 (18.2)	2.10 (1.17 to 3.79)	0.01	52 (34.7)	24 (16.7)	2.62 (1.57 to 4.38)	<0.001
Relaxation	67 (42.9)	13 (8.8)	8.14 (4.13 to 16.05)	<0.001	60 (40.0)	16 (11.1)	6.06 (3.86 to 9.52)	<0.001
Rest	45 (28.8)	8 (5.4)	7.22 (3.31 to 15.78)	<0.001	41 (27.3)	18 (12.5)	2.85 (1.63 to 5.01)	<0.001
Work	35 (22.4)	5 (3.4)	8.19 (3.75 to 17.86)	<0.001	26 (17.3)	12 (8.3)	2.22 (1.03 to 4.75)	0.04

The analyses made adjustments for baseline NPQ scores, duration of neck pain, age, gender, and city as a fixed effect and GP practice as a random effect using robust standard errors.

The above changes associated with the interventions at 6 and 12 months were analyzed to see if differences from usual care were also associated with changes in NPQ pain and disability scores (Table 6). A lessening of intervention effects, as shown in Table 6, indicates the possibility that the associated variables were acting as mediators. There is evidence of significant reductions in NPQ scores at 12 months related to changes in self-efficacy, ability to use what has been learnt, and changes in perceived stress.

**Table 6.** Impact of Factors Measured During the Intervention Period on Northwick Park Neck Pain and Disability Questionnaire Scores at 6 and 12 Months

	*6 months*		*12 months*	
*Characteristic*	*Difference in percentage points between acupuncture and usual care (95% CI)*	p	*Difference in percentage points between acupuncture and usual care (95% CI)*	p
Intervention effects (primary results)	−5.56 (−8.33 to −2.78)	<0.001	−3.92 (−6.87 to −0.97)	0.009
Self-efficacy				
Intervention	−3.31 (−5.62 to −0.99)	0.007	−2.28 (−5.28 to 0.73)	0.132
Change in self-efficacy	−3.01 (−3.75 to −2.26)	<0.007	−3.34 (−4.38 to −2.31)	<0.001
Ability to use what has been learnt				
Intervention	−5.57 (−7.98 to −3.16)	<0.001	−3.16 (−6.08 to −0.24)	0.035
Ability to use what has been learnt	−2.70 (−4.17 to −1.23)	0.001	−2.13 (−3.53 to −0.73)	0.004
Ability to make improvements in living self-care				
Intervention	−5.12 (−7.92 to −2.32)	0.001	−2.55 (−5.74 to 0.64)	0.113
Ability to make improvements in living/self-care	−3.90 (−7.17 to −0.63)	0.021	−2.35 (−5.73 to 1.03)	0.166
Summary of lifestyle change				
Diet				
Intervention	−4.95 (−7.48 to −2.42)	<0.001	−4.02 (−6.87 to −1.17)	0.007
Diet change	−2.59 (−6.58 to 1.40)	0.195	−0.33 (−5.18 to 4.52)	0.891
Exercise				
Intervention	−4.94 (−7.60 to −2.28)	0.001	−3.54 (−6.45 to −0.62)	0.019
Exercise change	−3.15 (−6.10 to −0.20)	0.037	−2.93 (−6.92 to 1.07)	0.145
Relaxation				
Intervention	−3.26 (−6.18 to −0.34)	0.030	−4.04 (−7.28 to −0.80)	0.016
Relaxation change	−6.22 (−10.57 to −1.87)	0.006	−0.04 (−4.54 to 4.47)	0.987
Resting				
Intervention	−4.64 (−7.49 to −1.80)	0.002	−4.08 (−7.02 to −1.15)	0.008
Rest change	−3.15 (−6.83 to 0.54)	0.092	0.22 (−4.29 to 4.72)	0.923
Work				
Intervention	−4.49 (−7.14 to −1.84)	0.002	−3.80 (−6.42 to −1.18)	0.006
Work change	−4.63 (−8.46 to −0.80)	0.019	−3.05 (−8.33 to 2.22)	0.246
Number of lifestyle changes				
Intervention	−3.44 (−6.29 to −0.59)	0.020	−3.58 (−6.82 to −0.35)	0.031
Number of lifestyle changes	−1.84 (−3.01 to −0.67)	0.003	−0.58 (−2.08 to 0.92)	0.436
Extent able to put into practice advice				
Intervention	−4.07 (−8.23 to 0.11)	0.056	−3.41 (−8.15 to 1.33)	0.152
Extent able to put into practice advice/teaching	−0.72 (−1.24 to −0.19)	0.009	0.02 (−0.00 to 0.05)	0.073
Extent changes have been helpful				
Intervention	−3.49 (−7.67 to 0.68)	0.098	−3.31 (−7.87 to 1.25)	0.149
Extent changes have been helpful	−1.08 (−1.77 to −0.39)	0.003	0.02 (−0.01 to 0.05)	0.153
Change in perceived stress				
Intervention	−5.76 (−8.01 to −3.51)	<0.001	−370 (−6.44 to −0.95)	0.010
Change in perceived stress	0.48 (0.05 to 0.92)	0.032	0.78 (0.30 to 1.25)	0.002

## Discussion

### Principal findings

Our key finding is that lifestyle advice based specifically on acupuncture theory leads to active patient engagement, which in turn is an important contributor to overall benefits over the longer-term. At 12-month follow-up, we found some evidence of congruence between practitioner-reported lifestyle advice and the actual lifestyle changes reported by patients receiving acupuncture related to diet, rest, and work. Compared with the patients receiving usual care alone, we found that patients in the acupuncture group made more lifestyle changes related to diet, exercise, relaxation, rest, and work and improved their self-efficacy, which is their ability to reduce their neck pain without resorting to medication. We found that patients in the acupuncture group improved their ability to use what they had learnt and increased their self-efficacy when compared with usual care alone and, in turn, these characteristics were found to be associated with significant reductions in NPQ pain and disability scores at 12 months. Overall, we have shown that the characteristics of the acupuncture provision in a pragmatic trial of acupuncture for chronic neck pain go well beyond acupuncture needling alone and specifically that pattern-related lifestyle advice has an impact on outcome.

### Strengths and weaknesses

As with any secondary analysis of data from a clinical trial that was powered for the main comparison, the desire to establish whether or not there were treatment components mediating outcomes may have been compromised by low statistical power. Moreover, we conducted multiple exploratory analyses, and if the results had been based on chance alone, we would have expected that 1 in 20 would be statistically significant. However, a strength of the study is that we prespecified the analyses that we conducted in a statistical plan, a plan that was finalized before conducting the primary analysis. Nevertheless, our results should be seen as exploratory.

Our analyses include both statistically significant and nonsignificant findings. The nonsignificant results are difficult to interpret as it is not clear whether the analyses are underpowered to find a small association or whether it means that no association exists. Further exploration of these relationships in other studies would help put these findings in context.

A limitation of our study is that we only recruited acupuncturists who practiced acupuncture according to traditional acupuncture principles. While this might be the predominant style of practice worldwide as well as in the United Kingdom,^2^ we caution against drawing conclusions about acupuncture when practiced using other treatment styles. We also note that there is some evidence of equivalence in outcomes across different styles and/or associated with different characteristics of acupuncture and of acupuncture practitioners.^18^ We acknowledge that as the trial was conducted in primary care in the United Kingdom, we must generalize with caution to other populations. Moreover, our results are relevant primarily to patients who, when recruited to the trial, had neck pain for an average duration of 6 years and 75% were taking medication (60% related to neck pain).

### Implications for research and practice

Pragmatic trials are not designed to separate out the extent that any putative benefits might be specific or nonspecific to the intervention. Our data are based on common practice among traditional acupuncture practitioners, whereby needling is one component of treatment, but so too is the provision of lifestyle advice.^2^ Our data show the way that lifestyle advice is specific to the diagnosis and is also associated with outcome and therefore cannot be labeled nonspecific. This point was made clearly by Paterson and Dieppe^1^ when they describe explanatory trial designs that attempt to divide an intervention into characteristic (specific) and incidental (placebo, nonspecific) elements as being neither meaningful nor feasible when evaluating complex nonpharmaceutical interventions, attempts that may generate false negative results. As an alternative, pragmatic designs can evaluate complex interventions and also prespecify and monitor the key variables that are thought to mediate outcomes. In this way, we can better understand the take up of lifestyle advice by patients and the strategies that might enhance patient benefits. Further research into the reasons why patients might, or might not, take up lifestyle advice and what strategies practitioners could use to enhance compliance with advice could help improve practice.

Our documentation on the reported symptoms of chronic neck pain, using the framework of traditional Chinese medical diagnostic principles, contributes to our understanding of neck pain. In particular, we noted the considerable overlap between physical and affective aspects of chronic neck pain, particularly stress, suggesting that acupuncture might be of special value when mental and emotional symptoms are involved along with physical symptoms. We suggest that it would be useful to extend the focus of acupuncture research beyond the treatment of single conditions and explore the impact of treatment where such comorbidities are common.

## Conclusion

We have reported in detail the intervention data for acupuncture in the ATLAS trial, including treatment variables that potentially mediated outcome. We have provided evidence that practitioners actively initiate the active engagement by the patient through targeted lifestyle advice, and that this makes a difference in the way patients learn to improve the way they live and care for themselves. This results in enhanced self-efficacy, which in turn is associated with greater reductions in pain and disability at 12 months. These data, when combined, provide a case to consider traditional acupuncture as not just a needle-related intervention but rather as a complex intervention that combines both acupuncture needling and acupuncture-related lifestyle advice that actively engages the patients in their own recovery.

## Supplementary Material

Supplemental data
