# Supplementary material for: Lifestyle Advice and Self-Care Integral to Acupuncture Treatment for Patients with Chronic Neck Pain: Secondary Analysis of Outcomes Within a Randomized Controlled Trial
Source: J Altern Complement Med. 2018 Mar 27;23(3):180–7. doi: 10.1089/acm.2016.0303 (PMC6266543; doi:10.1089/acm.2016.0303)
Supplement: Supplemental data [file Supp_Data.pdf]

Supplementary Data

Acupuncture Needling and Auxiliary Treatment Provided

Practitioners were asked to report the acupuncture points used for each patient in each treatment session. A total of 259 different individual acupuncture points were used at least once and 25,696 points were needled across all sessions. The 20 most commonly used acupuncture points are detailed in Supplementary Table S1. The two most common points, GB-20 and GB-21, are local neck pain points. Variations in the points reflected the diagnosis made, for example, patients diagnosed with Liver-related cluster more commonly received points designed to move stagnation, such as LIV-3, and those with a Spleen cluster more commonly received points for tonification, such as SP-6.

The 25,696 individual needle insertions equate to a mean of 14 insertions per patient per session (median = 14), with a range of 5 to 35. The two most common deepest needling depths reported were 1 cm (reported by 34% of practitioners) and 1.5 cm (33%). The majority of practitioners (90%) sought a *de qi* response when treating their patients, and the most common needle stimulation methods were even (63/160; 39%); tonifying, reducing, and even (44/160; 28%); and reducing and even (20/160; 13%). On average, practitioners retained the needles for around 20 min, which varied to some extent by patient, such that 73% of practitioners reported a minimum needle retention time as 15 to 20 min and 93% reported a maximum needle retention time of between 20 and 30 min. One hundred percent of practitioners used stainless steel needles to administer their treatments.

The use of auxiliary interventions based on the diagnosis was permitted within the treatment protocol guidelines. The most commonly used additional therapies were acupressure massage (used at least once on 109 of the 160 patients; 68%), followed by cupping (41/160; 26%) and the use of a heat lamp (40/160; 25%), moxibustion (38/160; 24%), electroacupuncture (7/160; 4%), and ear seeds (6/160, 4%).

Supplementary Table S1. Most Commonly Used Acupuncture Points

Rank	*Acupuncture point name*	*No. of patients used on (*n = *160),* n *(%)*	*No. of times point used over trial*	*Mean no. of times used within each course of treatment*	*% times used compared with all points on the whole trial*
1	Gallbladder 20	152 (95)	1175	7.7	4.6
2	Gallbladder 21	143 (89)	1090	7.6	4.2
3	Large Intestine 4	104 (65)	519	5.0	2.0
4	Liver 3	100 (63)	587	5.9	2.3
5	Bladder 10	91 (57)	390	4.3	1.5
6	Spleen 6	87 (54)	288	3.3	1.1
7	Small Intestine 3	84 (53)	395	4.7	1.5
8	Bladder 18	82 (51)	327	4.0	1.3
9	*Ah Shi* point	79 (49)	1291	16.3	5.0
10	Du 14	76 (48)	289	3.8	1.1
11	Bladder 23	75 (47)	371	4.9	1.4
12	Kidney 3	74 (46)	239	3.2	0.9
13	Bladder 13	73 (46)	266	3.6	1.0
14	Bladder 11	72 (45)	207	2.9	0.8
15	Stomach 36	71 (44)	270	3.8	1.1
16	*San Jiao* 5	69 (43)	299	4.3	1.2
17	Small Intestine 13	66 (41)	249	3.8	1.0
18	Bladder 15	64 (40)	271	4.2	1.1
19	Bladder 12	63 (39)	221	3.5	0.9
20	Bladder 60	59 (37)	159	2.7	0.6
